# Research progress on the immunomodulatory mechanism of acupuncture in tumor immune microenvironment

**DOI:** 10.3389/fimmu.2023.1092402

**Published:** 2023-02-14

**Authors:** Na Wang, Lu Zhao, Dou Zhang, Fanming Kong

**Affiliations:** ^1^ Department of Oncology, First Teaching Hospital of Tianjin University of Traditional Chinese Medicine, Tianjin, China; ^2^ National Clinical Research Center for Chinese Medicine Acupuncture and Moxibustion, Tianjin, China

**Keywords:** acupuncture, tumor immune microenvironment, traditional Chinese medicine, immunotherapy, cancer immunity

## Abstract

With the constantly deeper understanding of individualized precision therapy, immunotherapy is increasingly developed and personalized. The tumor immune microenvironment (TIME) mainly consists of infiltrating immune cells, neuroendocrine cells, extracellular matrix, lymphatic vessel network, etc. It is the internal environment basis for the survival and development of tumor cells. As a characteristic treatment of traditional Chinese medicine, acupuncture has shown potentially beneficial impacts on TIME. The currently available information demonstrated that acupuncture could regulate the state of immunosuppression through a range of pathways. An effective way to understand the mechanisms of action of acupuncture was to analyze the response following treatment of the immune system. This research reviewed the mechanisms of acupuncture regulating tumor immunological status based on innate and adaptive immunity.

## Introduction

The tumor immune microenvironment (TIME) is an important site of interaction between tumors and the human immune system. It mostly consists of tumor cells, immune cells, fibroblasts, endothelial cells, and other cells that provide favorable conditions for tumor proliferation and progression (1). Molecular biology and immunology found that there was a close relationship between the occurrence and development of malignant tumors and the immune microenvironment (2). Natural killer (NK) cells and cytotoxic T cells (CTL), which the immune system uses to eliminate tumor cells and provide the function of immune surveillance (3). Tumor cells secrete a range of immunosuppressive substances to alter the microenvironment as the number of tumor cell mutations increases. As a result, immune cells become polarized into immunosuppressive phenotypes like helper T cell 2 (Th2), regulatory T cell (Treg), bone marrow-derived inhibitory cells, and tumor-associated macrophages. These cells then secrete more immunosuppressive compounds, which allow immune cells to escape and accelerate rapid proliferation (4–6).

Both modern and traditional medicine are faced with the dilemma of actively exploring new ways to improve the survival time of patients with malignant tumors. Since the concept of comprehensive treatment was promoted, acupuncture was used in the traditional treatment methods owing to its unique advantage. Derived from traditional Chinese medicine (TCM), acupuncture has been inherited and carried forward through accumulated practice (7). A variety of needling techniques are used in acupuncture to cause physiological reactions that activate pathways in the peripheral and central nervous systems. Acupuncture typically entails inserting a tiny needle into acupoints and is occasionally used to treat thrusting and/or whirling (8). It features a broad range of capabilities with security and reliability.

Studies demonstrated that acupuncture improved symptoms through systemic conditioning of meridians and acupoints (9). It achieved systemic or local responses by local acupuncture around superficial tumors (10). Additionally, it had a positive therapeutic impact on a range of immunological disorders linked to tumors and could significantly enhance the capacity to fight infections (11). It was worth mentioning that mechanical stimulus activated regional cell processes and neuroreceptors (12). To achieve holistic regulation, it also controlled the release of related biomolecules (peptide hormones, lipid hormones, neuromodulators, neurotransmitters, etc.) (13). The immunomodulatory action of acupuncture was frequently paired with additional benefits (analgesia, antiaging, antistress, etc.), which worked together to strengthen the body’s immunity (14). Modern mechanism research showed that acupuncture took an anti-tumor role in promoting apoptosis of tumor cells, inhibiting proliferation, improving immunosuppression, and strengthening antioxidation (15, 16).

This paper summarized the mechanisms underlying acupuncture-mediated immune regulation from the perspective of innate immunity and adaptive immunity. A better understanding of these mechanisms would provide a scientific basis for the further clinical utilization of acupuncture in the treatment of malignant tumors.

## Acupuncture in regulating immune microenvironment

### Regulatory effect of acupuncture on innate immunity

As the initial line of body defense, innate immunity is an essential component of the immune system. It is also referred to as nonspecific immunity, which means a type of natural defense that can develop independently of antigen stimulation or develop gradually as a result of long-term ethnic evolution (17). Innate immunity can not only fend off the invasion of pathogenic microbes or toxic substances but also destroy pathogens through phagocytosis and disintegration. Additionally, it significantly influenced the beginning, development, and control of adaptive immunity (18). Studies confirmed that acupuncture had overall and dual regulatory effects on immune cells and molecules of the innate immune system.

#### Natural killer cells

NK cells are innate lymphocytes that are recognized in various organs (19). They possess typically cytotoxic mechanisms to lyse cancerous or virally infected cells. On the one hand, they directly release lytic granules which majorly consist of granzymes and perforin to induce the death of stressed cells. On the other hand, they also produce multiple tumor necrosis factor (TNF) superfamily members to cause cell apoptosis (20). Previous studies informed that acupuncture regulated NK cells in dual directions. When immunity was low (such as chronic stress and fatigue syndrome), acupuncture increased the number and activity of NK cells, and promoted NK cells to secrete immune factors (such as interferon (IFN)-γ, interleukin (IL)-10, granulocyte-macrophage stimulating factor, etc.) (21, 22). Besides, acupuncture reduced the number of NK cells when relieving pain (23). It activated NK cell receptor CD94 and tyrosine-protein kinase, promoted the expression of cytokines and adhesion factors, and decreased the expression of NK cell inhibitory pathway proteins.

The hypothesis of acupuncture immune enhancement was raised, which considered that acupuncture probably could prevent and kill tumor cells by increasing NK cells (24). In the cyclophosphamide-induced immunosuppressive model, electroacupuncture (EA) could increase the levels of lactate dehydrogenase and acid phosphatase, promote the proliferation of spleen cells induced by concanavalin A and lipopolysaccharide, and increase the killing toxicity of NK cells. Meanwhile, EA could promote the expression of cytokines IL-2, IL-12, TNF-α and IFN-γ, reduce the expression of IL-10, and improve the cyclophosphamide-induced immunosuppression by activating the NF-κB signaling pathway (25). Furthermore, EA combined with cisplatin effectively reduced the tumor volume of patients with stage IIb-IIIb cervical squamous cell carcinoma, and somewhat enhanced the proportion of NK cells in peripheral blood (26). Similarly, further research also demonstrated that EA increased the content of IFN-γ and enhanced the activity of NK cells (27).

#### Macrophages

Macrophages are the major component of the mononuclear phagocyte system, playing key roles in the innate immune system. They exist various functions and have significant effects on normal homeostasis and disease progression (28). According to the fundamental consensus of the macrophage activation phenotypes, macrophages are defined as pro-inflammatory (M1) and anti-inflammatory (M2) profiles. On the one hand, M1 macrophages have the capacity of starting and sustaining inflammatory responses, secreting pro-inflammatory cytokines, activating endothelial cells, and inducing the recruitment of other immune cells into the inflamed tissue; on the other hand, M2 macrophages promote the resolution of inflammation, phagocytose apoptotic cells, drive collagen deposition, coordinate tissue integrity, and release anti-inflammatory mediators (29). The disproportion of M1/M2 induced tumorigenesis and development, immune escape, subsequent metastasis, and drug resistance (30).

Regulating the phagocytic ability of macrophages and the secretion of immune factors were important cellular mechanisms for acupuncture to enhance immunity and treat diverse diseases. Acupuncture promoted the macrophage phenotype and IL-10 expression to mitigate the pain and inflammation of animal muscle. Further study demonstrated that acupuncture modified the macrophage phenotype of inflammatory muscle, and it could be the basic process causing the IL-10-induced analgesic and anti-inflammatory actions (31). In addition, EA was an adjuvant therapy for spinal cord injury based on decreasing the proportion of M1 macrophages, TNF-α, and IL-6 levels while enhancing the proportion of M2 macrophages, IL-10, and neurotrophin-3 (NT-3) expression (32). Tumor-associated macrophages play an important role in the functional composition of TIME, which usually stimulates proliferation, immunosuppression, and angiogenesis to promote tumor growth and metastasis (33). Therefore, the development of an antineoplastic method targeting macrophage polarization is also the focus of the current treatment.

#### Mast cells

Mast cell (MC) is a group of bone marrow-derived immune cells that differentiates and matures in peripheral tissues. MC plays an immunomodulatory role in both innate and acquired immune responses, thus affecting the progression and prognosis of related diseases (34). The infiltration of MC in TIME was closely related to the prognosis of malignant tumors (35). Previous studies confirmed that MC aggregated around tumors. It played a tumor-promoting or anti-tumor role in different types of tumors or in different processes of the same tumor, which affected tumor growth and metastasis mainly by affecting tumor angiogenesis, participating in immune regulation, and promoting tissue remodeling (36). There were many reports on the regulation of MC in acupuncture. MC aggregated in small blood vessels, nerves, and nerve endings of humans and rats along the meridian (37). Acupuncture stimulation could increase the aggregation and degranulation effect of MC. Histamine produced by the degranulation of MC acted on blood vessels and triggered meridian sensing (38). Besides, acupuncture regulated the function of MC in both directions and it alleviated the abnormal degranulation of MC under pathological conditions (39). Acupuncture relieved MC degranulation and promoted synaptic-like function, which was an important immune mechanism for the treatment of allergic diseases and inflammatory diseases (40).

#### Microglia

Microglia is an important type of innate immune cell in the central nervous system, which is the main effector in the process of inflammation progression (41). Acupuncture was used to treat neurodegenerative disorders, central traumatic diseases, and neuropathic pain by regulating the microglia (42–44), which was widely involved in a variety of pathological processes. Increasing evidence suggested that microglial activation was critical for neurogenesis, angiogenesis, and synaptic remodeling. It could move to the site before tumor metastasis, induce local and systemic immunosuppression, promote tumor angiogenesis, reorganize surrounding tissue, promote matrix remodeling and tumor invasion, and take a vital part in tumor brain metastasis (45–47). The mechanism of chemotherapy-induced peripheral neuropathy was considered as persistent activation of spinal cord microglia through strengthening TREM/DAP12 signaling (48). On the contrary, the therapeutic mechanism of action of paclitaxel-induced pain hypersensitivities has been confirmed that EA effectively and persistently suppressed TLR4 signaling and TRPV1 upregulation in DRG neurons, which further resulted in reduced spinal glia activation (49). Furthermore, it was mutually corroborative that the feasible mechanism was associated with the inhibition of the activated TLR4/NF-κB signaling pathway (50).

#### Other innate immune cells

Neutrophils, which originate from myeloid precursors, are crucial components of white blood cells and the first responders of the innate immune system against extracellular pathogens and wound healing (51). Recent studies demonstrated that neutrophils were widely involved in the occurrence, development, migration and invasion of tumors (52), and neutrophils with different phenotypes played a significant part in the formation of tumor immunosuppressive microenvironment (53). In the process of innate immunity, neutrophils are gathered and activated at the inflammatory site. They activated signal transduction pathways by releasing inflammation-related factors such as reactive oxygen species (ROS) and cytokines, which formed a series of cascade reactions to regulate inflammation and immunity (54, 55). Acupuncture bidirectionally regulated neutrophils. It increased the number and activity of neutrophils when inflammatory reactions occurred (56), while it down-regulated the number and activation rate of neutrophils in stressful conditions or allergic diseases to counteract stress damage (57).

Dendritic cell (DC) is a rare group of immune cells in tumor tissues, which regulates immune signals by ingesting and presenting antigens to T cells, contacting directly between cells, and providing cytokines (58). The regulation of DC function is significantly influenced by environmental factors. The initiation of antigen-characteristic immunity and tolerance can be regulated by cell surface and intracellular cytokine receptors, pathogen-related molecular models and injury-related molecular models (59). As an important antigen-presenting cell, DC is commonly in a state of immunosuppression or immune tolerance in TIME. The role of different DC cell subsets and their immunomodulatory mechanism is of great significance for clinical treatment. Conventional DC (cDC) 1 induced the anti-tumor immune response of killer T cells and improved the survival rate of cancer patients, while cDC2 induced the anti-tumor immune function of CD4^+^ T cells (60). Under the intervention of acupuncture, DC tends to gather at the acupoint after activation from the surrounding tissue. It is speculated that the related mechanism may be caused by the neurogenic response of the skin around the acupoint after acupuncture stimulation (61, 62). The research on the relationship between acupuncture and DC is still in the stage of exploration and development, which needs to be supported by further research data.

### Regulatory effect of acupuncture on adaptive immune cells

Adaptive immunity includes T-cell-mediated cellular immunity and B-cell-mediated humoral immunity. The main force involved in anti-tumor adaptive immunity is T lymphocytes, which mainly include CTL and Treg cells (63). As the main member of anti-tumor immunity, CTL can recognize the antigen complex on the surface of tumor cells and secrete granzyme and perforin to clear tumor cells. However, under the tumor background, immunosuppressive cytokines (such as IL-10, IL-4, CX3CL, etc.) and negative costimulatory molecules (such as PD-1/PD-L1, CTLA-4/CD80/CD86, etc.) greatly limit the proliferation and activity of CTL and even cooperate with tumor extracellular matrix to exclude CTL from the tumor parenchyma, making it unable to contact tumor cells (64, 65). Treg cells can produce immunosuppressive cytokines (such as TGF-β and IL-10) and CTLA-4, which hinder the activation and expansion of CTL (66, 67).

#### Acupuncture on cellular immunity

Cellular immunity is the main immune response in human autoimmune mechanisms. The immune function of patients with malignant tumors is related to the imbalance of the proportion and quantity of T cells. The T-cell-mediated immune response is characterized by the presence of an inflammatory response dominated by monocyte infiltration and/or specific cytotoxicity. Acupuncture has been confirmed that regulated the differentiation of T-cell subsets and promoted the drift of Th1/Th2 and Th17/Treg toward Th2 and Treg, respectively (68). Thus, it led to a reduction in pro-inflammatory factors (such as IL-2, IL-12, and IFN-γ) and an increase in the expression of IL-10. It decreased serum antibodies and nitric oxide and also affected the levels of inflammatory signaling molecules (such as ERK, NF-κB, AP-1, p38, etc.) (69). This modulation of T cell subsets and their associated inflammatory response factors was considered an important cellular immune mechanism for acupuncture in the treatment of allergic diseases and nonspecific inflammatory diseases (70–73). Besides, acupuncture regulated the expression of FAS/FAS-L in the thymus, increased the apoptosis rate of T cells in peripheral blood and mediated T cell tolerance, which had therapeutic effects on rheumatoid arthritis (74).

As for the treatment of cancer-related diseases, the absolute account of T cell subsets and anti-tumor response were closely related to the prognosis of tumor patients. Preclinical studies demonstrated that more CD4^+^ and CD8^+^ cells were likely responsible for the extended survival time and successful treatment of tumors (75–77). Additionally, in rat models with bone cancer pain, EA reduced mechanical allodynia even though its analgesic effectiveness was inferior to morphine. Contrary to morphine, the T cell proliferation, plasma IL-2 level, as well as the proportions of CD3^+^CD4^+^ and CD3^+^CD8^+^ T cells in the EA group were significantly increased (78). The analgesic and immunomodulatory effects of EA may have the same mechanism through an opioid-mediated pathway and need to be further investigated. In patients with gastric cancer, acupuncture compound general anesthesia reduced the negative impacts on immune function based on obviously decreased levels of CD3^+^, CD4^+^ and CD4^+^/CD8^+^ after subtotal gastrectomy (79). Similarly, in patients following gastrointestinal tumor resection, EA compound general anesthesia had a satisfactory anesthetic effect and immunomodulatory function, which had lower levels of TNF-α, IL-6, and IL-1β and decreased levels of CD3^+^, CD4^+^ and CD4^+^/CD8^+^ (80). As for cancer-related fatigue, acupuncture was beneficial to improve the symptoms of fatigue and promote the absolute counts of CD3^+^, CD4^+^, CD8^+^ and CD4^+^/CD8^+^ (81). Saam acupuncture was consistent with the above outcomes that had a statistical expression in CD3^+^, CD8^+^ and T-cell subsets (82). In conclusion, acupuncture is capable of effectively increasing the level of T cells in TIME, especially the absolute number of cytotoxic T cell subsets, which is an important tool to enhance the effectiveness of tumor immunotherapy.

#### Acupuncture on humoral immunity

The proliferation, differentiation and antibody secretion of B cells are the basic processes of humoral immunity. Acupuncture was demonstrated that had a dual regulatory effect on the production of antibodies by B cells (83). When immune intolerance occurs in the organism, antibodies are frequently found in the serum of overexpression. Acupuncture could reduce the content of serum immunoglobulins (IgG, IgA, IgM) in allergic diseases and non-inflammatory diseases (84–86). Conversely, it could increase the production of IgG and IgM when the humoral immune response was insufficient. During surgery, EA seemed to lessen the immunosuppression of both the humoral and cellular components. When comparing immunoglobulin levels postoperatively, the IgA levels were significantly higher in the EA group compared to the pure-drug group. Under the same circumstances as general anesthetic alone, needles combination anesthesia partially attenuated perioperative immunosuppression (87). In addition, acupuncture had indirect regulatory effects on humoral immunity by affecting antigen-presenting cells, the release of immune factors and the generation of complement.

### Outlook and summary

Malignant tumors produce or secrete some immunosuppressive substances to evade the role of the immune system, to expect the normal growth and proliferation of tumor cells (88). Acupuncture has a sufficiently dual regulating effect and can improve immune function. The mechanism of tumor elimination by restoring the body’s immune ability is consistent with the concept of “nourishing positive accumulation and eliminating cancer by itself” in TCM (89). The formation and dynamic changes in TIME involve many different types of cells and multiple signaling pathways, which are similar to the multitarget and bidirectional regulation of immunity in TCM (90).

Discussing the relationship and mutual influence of acupuncture on TIME are current research hotspots, but the accuracy, depth and breadth of the research are not enough. From the retrieved literature, most of the mechanism studies were still focused on the mechanism of immune function. Moreover, the research was still based on the dose-effect study of acupuncture on the tumor, and there was little discussion on the internal mechanism and pathway of the tumor. Therefore, the content of this article is both logical and speculative. Future research should explore the mechanism of acupuncture on TIME more deeply from the aspects of signal pathway with modern biology and technology. This will better provide a theoretical basis and standardized treatment for clinical treatment.

## Author contributions

All authors contributed to the study’s conception and design. Material preparation, paper collection and analysis were performed by FK and NW. The first draft of the manuscript was written by NW and all authors commented on previous versions of the manuscript. All authors contributed to the article and approved the submitted version.
